# Effects of choral singing versus health education on cognitive decline and aging: a randomized controlled trial

**DOI:** 10.18632/aging.202374

**Published:** 2020-12-18

**Authors:** Lei Feng, Rafael Romero-Garcia, John Suckling, Jasmine Tan, Anis Larbi, Irwin Cheah, Glenn Wong, Maurine Tsakok, Bernard Lanskey, Darius Lim, Jialiang Li, Joanna Yang, Benjamin Goh, Tristan Gwee Chen Teck, Allan Ho, Xiu Wang, Jin-Tai Yu, Can Zhang, Crystal Tan, Michelle Chua, Junhua Li, John J Totman, Caroline Wong, Marie Loh, Roger Foo, Chay Hoon Tan, Lee Gan Goh, Rathi Mahendran, Brian K. Kennedy, Ee-Heok Kua

**Affiliations:** 1 Department of Psychological Medicine, Yong Loo Lin School of Medicine, National University of Singapore, Singapore; 2 Healthy Longevity Translational Research Program, Yong Loo Lin School of Medicine, National University of Singapore, Singapore; 3 Centre for Healthy Longevity, National University Health System, Singapore, Singapore; 4 Department of Psychiatry, University of Cambridge, UK; 5 Department of Psychology, Goldsmiths, University of London, UK; 6 Biology of Aging Laboratory, Singapore Immunology Network, Singapore; 7 Department of Biochemistry, Yong Loo Lin School of Medicine, National University of Singapore, Singapore; 8 Maurine Tsakok Inc, Singapore; 9 Yong Siew Toh Conservatory of Music, National University of Singapore, Singapore; 10 Darius Lim, Voices of Singapore Choral Society, Singapore; 11 Department of Statistics and Applied Probability, Faculty of Science, National University of Singapore, Singapore; 12 Presbyterian Community Services, Singapore; 13 NTUC Health Co-operative Limited, Singapore; 14 Beijing Chui Yang Liu Hospital, Beijing, PR China; 15 Department of Neurology and Institute of Neurology, Huashan Hospital, Shanghai Medical College, Fudan University, Shanghai, China; 16 Genetics and Aging Research Unit, McCance Center for Brain Health, MassGeneral Institute for Neurodegenerative Disease, Department of Neurology, Massachusetts General Hospital and Harvard Medical School, Charlestown, MA 02129, USA; 17 School of Computer Science and Electronic Engineering, University of Essex, UK; 18 Clinical Imaging Research Centre, Yong Loo Lin School of Medicine, National University of Singapore, Singapore; 19 Lee Kong Chian School of Medicine, Nanyang Technological University, Singapore, Singapore; 20 Department of Epidemiology and Biostatistics, Imperial College London, London W2 1PG, UK; 21 Cardiovascular Research Institute, National University Health Systems, Singapore; 22 Genome Institute of Singapore, Singapore; 23 Department of Pharmacology, Yong Loo Lin School of Medicine, National University of Singapore, Singapore; 24 Division of Family Medicine, Yong Loo Lin School of Medicine, National University of Singapore, Singapore; 25 Academic Development Department, Duke-NUS Medical School, Singapore; 26 Department of Biochemistry, Yong Loo Lin School of Medicine, National University of Singapore, Singapore, Singapore; 27 Department of Physiology, Yong Loo Lin School of Medicine, National University of Singapore, Singapore, Singapore; 28 Singapore Institute for Clinical Sciences, Agency for Science, Technology and Research (A*STAR), Singapore, Singapore

**Keywords:** choral singing, health education, cognitive decline, biological markers, randomized controlled trial

## Abstract

We conducted a randomized controlled trial to examine choral singing’s effect on cognitive decline in aging. Older Singaporeans who were at high risk of future dementia were recruited: 47 were assigned to choral singing intervention (CSI) and 46 were assigned to health education program (HEP). Participants attended weekly one-hour choral singing or weekly one-hour health education for two years. Change in cognitive function was measured by a composite cognitive test score (CCTS) derived from raw scores of neuropsychological tests; biomarkers included brain magnetic resonance imaging, oxidative damage and immunosenescence. The average age of the participants were 70 years and 73/93 (78.5%) were female. The change of CCTS from baseline to 24 months was 0.05 among participants in the CSI group and -0.1 among participants in the HEP group. The between-group difference (0.15, *p*=0.042) became smaller (0.12, *p*=0.09) after adjusting for baseline CCTS. No between-group differences on biomarkers were observed. Our data support the role of choral singing in improving cognitive health in aging. The beneficial effect is at least comparable than that of health education in preventing cognitive decline in a community of elderly people. Biological mechanisms underlying the observed efficacy should be further studied.

## INTRODUCTION

Cognitive function declines with increasing age. This universal phenomenon affects the majority of our elders. Impaired cognitive function represents a major obstacle for healthy, functional, productive and successful aging. With rapidly-aging populations, effective interventions are critical for maintaining good cognitive function and preventing age-related cognitive decline. Candidates for interventions require careful evaluation in randomized controlled trials (RCT) [1].

There is increasing worldwide attention to the preventive role of lifestyle and behavioral modifications that could reduce the risk for cognitive decline. Encouraging results are now emerging from a large trial in Finland on the efficacy of a multi-domain intervention program [2] in improving cognitive function. In contrast, two large preventative trials [3, 4] did not show hypothesized benefits on cognitive outcomes. Although interest in multi-domain interventions is currently high, we believe that single interventions also offer the potential to improve cognitive health for aging populations. Choral singing is a novel and promising candidate that has not yet been assessed by a well-designed RCT.

People engaging in lifelong music-making have been found to have better cognitive outcomes later in life. A cross-sectional study showed that older adults who sang or played a musical instrument had higher crystallized intelligence, higher processing speeds, better letter fluency and better learning ability [5]. Both amateur and professional singers and musicians have brain features younger than their chronological age, suggesting that music-making has an age-decelerating effect [6]. Singing may therefore be an engaging and effective way to prevent age-related cognitive decline.

Singing programs have been found to improve cognition and psychological well-being in individuals with dementia [7]. A small pilot study found improved psychomotor processing speed and reduced neuropsychiatric symptoms of dementia after 6 months of weekly singing sessions [8]. An RCT involving 89 dementia patient-caregiver dyads demonstrated that 10 weeks of singing significantly improved mood, orientation, and remote episodic memory, and to a lesser extent, attention, executive function and general cognition [9]. Furthermore, choral singing interventions for non-demented elderly have demonstrable psychological benefits, including improved morale, decreased loneliness, improved mental health-related quality of life (QOL), and reduced levels of anxiety and depression compared to controls [10]. Choral singers also report higher physical health-related QOL than matched controls [11]. Older adults in a choral singing intervention not only had higher self-ratings of health, but also better objective health outcomes such as fewer visits to seek medical advice and treatment [12]. Choral singing’s cognitive benefits for non-demented older adults have not yet been studied, though the findings from other singing interventions are promising. An RCT found that a music intervention involving rhythmic exercises and improvisational singing improved cognition and executive function in healthy older adults [13] and a small pilot study found that 12 weeks of group singing improved memory and verbal fluency, as well as lung function [14].

In this RCT, we hypothesized that choral singing would improve cognitive health and/or reduce cognitive decline in elderly with high risk of dementia. Additionally, choral singing would be more effective than health education program (the control arm) at improving cognitive health and/or reducing cognitive decline in elderly with high risk of dementia. Lastly, we hypothesized that the improvement of cognitive health (measured by composite cognitive test scores) will be accompanied by change in biological measures of brain ageing derived from magnetic resonance imaging, immune markers of ageing and markers of oxidative damage.

Therefore, we compared the effect of a two-year, weekly choral singing intervention (CSI) on cognitive changes with a structured health education program (HEP) conducted with the same duration and frequency. Using an active comparator instead of a non-interventional control allows for the evaluation of potential superiority and non-inferiority of CSI against HEP. If effective, CSI could be scaled-up for communities or populations in countries with existing resources and a cultural interest in choral music. The primary outcome was the change in cognitive function during intervention period. We also measured secondary biological outcome variables, including brain magnetic resonance imaging (MRI) metrics, blood markers of immunosenescence and peripheral markers of oxidative damage [15].

## RESULTS

**Figure 1:**
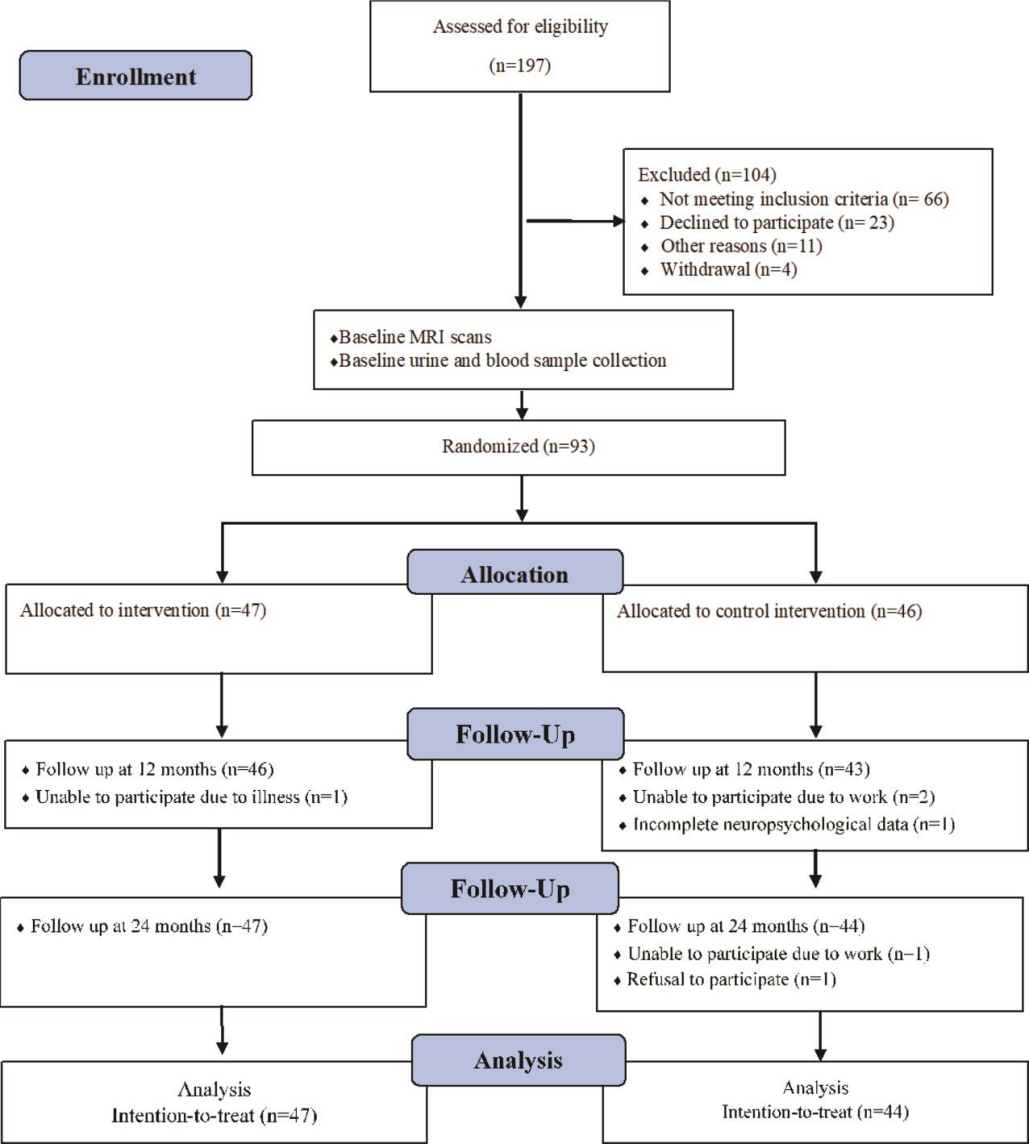
**Flow chart of participants through trial.**

Between 25^th^ August 2015 and 23^rd^ October 2015, 197 people were screened for eligibility assessment, 104 were excluded and 93 were randomized: 47 to the CSI arm and 46 to the HEP arm. Follow-up assessments at 12 and 24 months were completed for a total of 89 and 91 participants respectively (Figure 1). There were no harms or unintended events in either CSI or HEP groups during the two-year intervention period. Baseline characteristics of randomized participants are shown in Table 1; a T-test was conducted and the difference in all variables (i.e. age, education, Composite Cognitive Test Score (CCTS), Singapore Modified Mini-Mental State Examination (SM-MMSE) and Geriatric Depression Scale (GDS)) were not statistically significant. Hence, both CSI and HEP groups were comparable.

**Table 1:** Baseline demographic and clinical characteristics of randomized participants.

**Characteristics**	**Health education program (n=46)**	**Choral singing intervention (n=47)**
Mean (SD) age (years)	69.4 (5.3)	71 (5.7)
Women	37 (80.4)	36 (76.6)
Education level:		
No formal education	4 (8.7)	10 (21.3)
Primary school and below	29 (63)	25 (53.2)
Secondary school and above	13 (28.3)	12 (25.5)
Mean (SD) education (years)	6.7 (3.7)	6.1 (4.4)
Marital status:		
Single	1 (2.2)	2 (4.3)
Married	25 (54.3)	26 (55.3)
Divorced or separated	3 (6.5)	4 (8.5)
Widowed	17 (37)	15 (31.9)
Living alone	7 (15.2)	7 (14.9)
Hypertension	20 (43.5)	28 (59.6)
Diabetes mellitus	11 (23.9)	14 (29.8)
Heart diseases	2 (4.3)	1 (2.1)
Mean (SD) CCTS	0.1 (0.75)	-0.1 (0.6)
Mean (SD) SM-MMSE score	28.1 (1.8)	28.2 (1.9)
Mean (SD) GDS score	1.5 (2.2)	2.5 (3.3)

**Figure 2:**
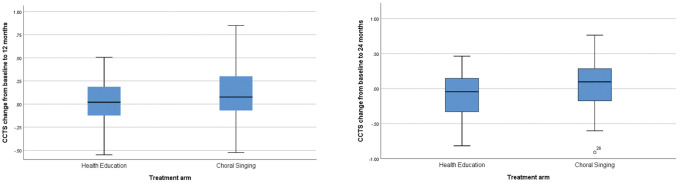
**Box plots of raw data for CCTS change from baseline to 12 and 24 Months. Data are median (central line), interquartile range (box margins), adjacent values (whiskers), and outlier (dots).**

The mean CCTS of participants in the CSI and HEP groups increased 0.1 and 0.02 respectively at 12 months. At the end of 24 months, mean CCTS increased 0.05 for participants in the CSI arm while the score of participants in the HEP arm decreased 0.1. The difference in absolute changes of CCTS (point estimate 0.15, 95% confidence interval 0.01 to 0.3) was statistically significant at 24 months at *p*<0.05. Figure 2 shows the raw data for absolute changes of CCTS. The differences narrowed (point estimate 0.12, 95% confidence interval -0.02 to 0.26, *p*=0.09) when the models were adjusted for baseline CCTS; the adjusted mean value of absolute change of CCTS for CSI and HEP groups was 0.036 and -0.083 respectively at 24 months (Table 2).

**Table 2:** Estimates of treatment effect on CCTS

**Dependent variable**	**12 months**					**24 months**				
	**Health education**		**Choral singing**		**p value**	**Health education**		**Choral singing**		**p value**
	**No**	**Mean (SE)**	**No**	**Mean (SE)**		**No**	**Mean (SE)**	**No**	**Mean (SE)**	
**CCTS change from baseline**	44	0.02 (0.03)	45	0.1 (0.04)	0.14	44	-0.1 (0.05)	47	0.05 (0.05)	0.042
Adjusted for baseline CCTS*	44	0.004 (0.05)	45	0.08 (0.04)	0.18	44	-0.083 (0.05)	47	0.036 (0.05)	0.091*

*Observed power=0.394.

There were no significant between-group differences on percentage changes of CCTS at 12 months and 24 months (data not shown). We also did not find a statistically significant main effect of treatment type *(p=*0.37) and the interaction term time* allocation *(p=*0.22) from a Linear Mixed-effects (LME) model that consisted of intercept, fixed effects from group allocation, time, group*time and random intercepts and random slopes for each of the participants. Similar results from LME models were found when we excluded the interaction term and added age, gender and education as fixed effects (Supplementary Figure 2, Supplementary Data Additional Tables and Figures).

**Figure 3:**
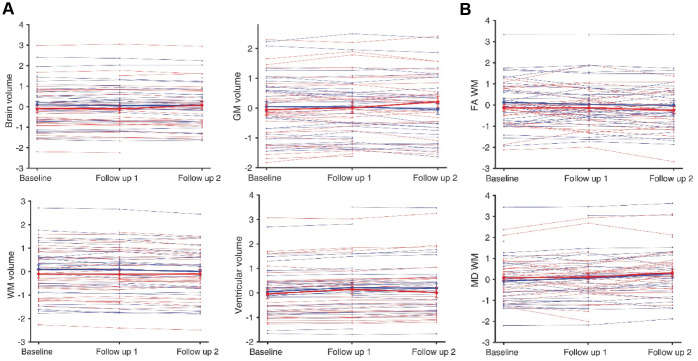
****Total Brain, GM, WM and ventricular volume (**A**), and FA and MD associated with WM of trial participants (**B**). Thin lines represent individual participants whereas thick lines illustrate the average of the choral singing (blue, 37 participants) and health education (red, 34 participants) groups. Values are normalized to sample average baseline. Error bars represent the standard error of the mean.

There were no significant effects of the CSI on any of the brain metrics. Effect sizes were small for most metrics, with only grey matter (GM) volume, white matter (WM) fractional anisotropy (FA) and WM mean diffusivity (MD) showing slightly larger effect sizes of between 0.22 and 0.26 (Figure 3, Table 3). As expected, there was a significant main effect of time in the LME model on most of brain metrics with significant changes in total brain, GM, WM, ventricular and hippocampal volumes, as well as MD for GM and WM (Figure 3, Table 3 and Supplementary Figure 3, Supplementary Data Additional Tables and Figures). The age of the participants at baseline was also significantly associated with MD, ventricular and hippocampal volume. While gender had a significant effect on most volumetric measures derived from the T1-weighted scan, there were no significant gender effects on the FA and MD values derived from diffusion tensor imaging. Education had significant effects on brain and WM volume.

**Table 3:** P-values associated with the fixed terms for brain MRI metrics.

	**Intercept**	**Intervention groups**	**Time**	**Age**	**Gender**	**Education**	**Group effect size**
Brain Volume	0.105	0.154	<0.001	0.184	<0.001	0.050	0.013
Ventricular Volume	0.116	0.976	<0.001	0.036	0.089	0.993	0.169
GM Volume	0.133	0.549	0.044	0.188	<0.001	0.091	0.253
WM Volume	0.098	0.063	<0.001	0.085	0.005	0.004	0.106
Hippocampal Volume	0.002	0.802	0.031	<0.001	0.043	0.847	0.165
FA WM	0.290	0.181	0.121	0.174	0.974	0.094	0.225
FA Cortical	0.851	0.984	0.999	0.571	0.170	0.239	0.049
MD WM	<0.001	0.183	0.020	<0.001	0.638	0.561	0.243
MD Cortical	0.036	0.375	0.001	0.013	0.619	0.188	0.173

There were no significant differences between the CSI and HEP groups on levels of the oxidative biomarker levels (Supplementary Figure 4, Supplementary Data Additional Tables and Figures). No significant differences were observed between CSI and HEP groups on markers of immunosenescence, including the frequencies of hematopoietic stem cells (HSCs), CD4:CD8 ratios, naïve and effector memory RA (TEMRA) CD8 T-cells, naïve CD4 T-cells and recent thymic emigrants (RTEs), senescent T-cells expressing CD57, PD-1 and KLRG1 (Supplementary Figure 5, Supplementary Data Additional Tables and Figures), suggesting that both have a similar impact on these measures.

## DISCUSSION

In this interim analysis of an RCT, we assessed the effects of CSI compared with a HEP on cognitive decline during a two-year intervention period. We observed an increase in the mean CCTS among participants in the choral singing group and a decrease in the mean CCTS among participants in the health education group. We did not find differences on measures of brain aging, oxidative damage and immunosenescence. The findings on CCTS changes suggest it is possible that choral singing is superior to health education in promoting cognitive health in older people, but a definitive conclusion cannot be drawn given inadequate statistical power and inconsistent findings using different analysis approaches. Further research should examine the efficacy with larger sample sizes, against existing interventions as well as empty controls where feasible.

It is important to note that there was a between-group difference on CCTS at baseline, though the difference was not statistically significant. Since participants in the CSI group had lower CCTS at baseline, theoretically they had an advantage when change scores are used as the outcome variable because of negative correlations between baseline and change scores, commonly known as the “regression to the mean” phenomenon. When baseline CCTS values were controlled for, the between-group difference on absolute changes in CCTS became non-significant, although the effect size was still considerable.

Based on this set of data, we are confident in concluding that CSI is at least as effective in delaying cognitive decline compared to a structured HEP targeting known risk factors of dementia such as hypertension, obesity, smoking, depression, physical inactivity, diabetes, and social isolation [16]. The proposed superiority of choral singing over health education needs to be further examined and confirmed with larger sample sizes. We will be able to draw more definitive conclusions on the proposed superiority when the sample size is increased by including the planned future cycles in the trial and potential extension of the study.

Although most of brain markers showed the expected decline associated with aging, the group allocation had no significant effect on any of the descriptors considered here, suggesting that both interventions are similarly effective (or ineffective) in preserving brain integrity. Nevertheless, previous cross-sectional and longitudinal studies based GM/WM volume and FA markers performed in cohorts with similar sample sizes have revealed that whole-brain volumetric and diffusion markers are sensitive to age decline [17, 18] and group intervention [19, 20] in the Chinese population. The absence of an association here may suggest that whole-brain markers are not specific enough to detect potential non-overlapping effects of choral singing and health education. WM volume showed a trend toward a group effect (*p*=0.06) while FA and MD located at WM had the largest effect sizes of all metrics considered. This suggests that understanding WM microstructural and organizational divergences could be critical for characterizing the differential impact that HEP and CSI may have in the brain. Further investigation of diffusion imaging data using morphometric analysis, Region-Of-Interest approaches or Tract-based Spatial Statistics (TBSS) will be necessary to determine whether the potential benefits of choral singing over health education are restricted to localized brain regions and specialized circuits.

The elevations in biomarkers of oxidative damage have long been known to be a pathological factor in neurodegeneration and cognitive decline [21, 22]. Measuring stable biomarkers of oxidative damage in human biofluids can give valuable insights into the sum of all damage-driven biological processes and can correlate with onset of disease and ‘unhealthy’ paradigms [23]. However, there were no significant between-group differences on peripheral markers of oxidative damage in our study. Unfortunately, oxidative stress (and hence levels of damage biomarker) are subject to a wide range of factors (genetic, environmental, dietary, etc.), which may give rise to a higher degree of variability. This may be overcome by an increase in sample size.

Empirical evidence has revealed a role of the nervous system in regulating immunological responses [24–26]. Since music appreciation and production exert psychophysiological influence over the autonomous nervous system [27], choral singing could theoretically modulate immunological imbalances that accumulate with age. In single but separate sessions, Kreutz et. al. observed increased S-IgA levels in participants immediately after singing and listening to choral music [28], supporting the idea that choral singing can trigger short-term immunological responses. However, in this study we did not find favourable effects of CSI on the immunosenescence profiles of elderly participants when compared with HEP. Although we did not specifically study changes in immunoglobulin levels, transient immunological modulations are likely to have been overlooked because blood collection was organised annually as well as during occasions that were isolated from choir rehearsals. The observed fluctuations in the immunological markers measured in this study were minimal. Thus, with reference to reports which suggest that immunosenescence is slow but progressive [29, 30], it is possible that the choral singing intervention might have contributed to the preservation of memory lymphocyte precursors during the two-year intervention period, but its impact may not be immediately noticeable.

The strengths of our study include low drop-out rate and repeated measurement of cognitive function, brain imaging and markers of biological aging. However, we did not include a non-intervention control group so the true effects of the CSI on various measures in comparison with the natural history of age-related changes are unknown. Also, since CSI was provided as an integrated package, we are not able to tease out relative contributions of the putative cognitive enhancing components such as exposure to music, making music, and interactions in a group setting. Multiple intervention arms (for example: listening to music vs. singing alone vs. choral singing) could be considered in future studies, but such designs increase costs and the complexity of trial logistics. Another obvious limitation is the relatively small sample size, providing only 39% power for the observed difference on absolute changes of CCTS at 24 months, when baseline CCTS was adjusted for.

Our study is the first randomized trial in the world that systematically assessed the effects of singing on cognitive decline in aging and the potential effects on brain imaging, immune system and oxidative damage markers. Our findings from the very first RCT on this topic suggest that choral singing is a potentially useful intervention for the promotion of cognitive health in aging. Choral singing is a safe and enjoyable activity, and is likely to be embraced by the community. Policy makers may consider promoting choral singing for healthy and active aging of seniors in the community. This is especially relevant for countries where existing resources are available.

## MATERIALS AND METHODS

### Study design

This was a single site, parallel-group, randomized active-controlled trial to assess the efficacy and mechanisms in delaying cognitive decline of CSI versus HEP for two years. The study was approved by the National University of Singapore Institutional Review Board (NUS 2508). All participants gave written informed consent. Four cycles of the interventions are planned, however, only available data from the first cycle are reported as a planned interim analysis. Details of the study protocol have been described previously [15].

### Participants

We recruited 93 community-living elderly aged 60-84 years. All participants fulfilled at least one of the following criteria: (1) self-reported subjective cognitive complaints; or (2) early cognitive impairment defined by age- and education-standardized Z-scores in the range of -1.5 to 0 for at least five out of ten cognitive test scores (details on the tests are provided in the section on cognitive outcomes); or (3) having at least two risk factors of dementia from: family history of dementia, low physical activity, low social engagement, heart disease, transient ischemia attack (TIA), diabetes, history of head injury, living alone and having depressive symptoms. Exclusion criteria were: Clinical Dementia Rating (CDR) global score >0, terminal illness, stroke, aphasia, marked hearing impairment, or participation in an ongoing interventional study. A modified Singapore version of the CDR [31] was administered for the assessment of dementia severity and calculation of CDR global score.

### Study treatments

The CSI was held weekly for two years at the Yong Siew Toh Conservatory of Music, National University of Singapore. Each session was one-hour long. Each session incorporated the musical, social, and physical aspects of choral singing. The focus during the sessions was to educate singers to understand the concept of sound, the mechanics of the singing voice, and to differentiate good from bad singing. Participants were then taught more in-depth skills of correct, healthy vocal production using good breathing techniques, support and listening skills. Later in the program, participants learned to sing in different parts. The parts were taught aurally and slowly, helping each singer to understand how they represent different lines in the musical harmony at any point in a two- or more-part musical piece. Professional musicians facilitated the sessions in the interventions. We also included performances as part of the intervention program, the purpose of which was to promote motivation and a sense of purpose, pride and accomplishment.

The HEP was held weekly for two years at the Training and Research Academy at Jurong Point (TaRA@JP), Singapore. Each session was one-hour long. The sessions consisted of short talks on a health-related topic (diabetes, physical activities, healthy eating, depression, etc.) followed by group activities that required memory work and the acquisition of certain skills, but did not involve singing. Physical activity was incorporated with 10 minutes of exercise at each session. Family physicians, specialist clinicians and community nurses facilitated the interventions.

Both CSI and HEP involve cognitively demanding tasks such as memorizing new materials. Both CSI and HEP were delivered in a group setting as a group activity. Hence, there are similarities between the two intervention programs on common elements such as social interactions, a sense of belonging and the building of friendships over time. Both interventions may enhance levels of social engagement among participants while our observational study supports an association between higher social engagement and better cognitive health in aging [32].

### Data collection

Trained research nurses collected questionnaire data through face-to-face interviews at TaRA@JP, Singapore. We used the GDS [33, 34] for depressive symptoms and the SM-MMSE [35] as a global measure of cognitive function. Trained research assistants conducted the neuropsychological assessment to collect cognitive data at baseline, 12 months, and 24 months. Training to all research staffs was provided by a single instructor (LF) to ensure consistency and data quality.

### MRI acquisition and processing

MRI data depicting brain structure were collected using a 32-channel head coil on a 3T Prisma Siemens MR scanner at the Clinical Imaging Research Centre (CIRC) at the National University of Singapore (NUS). High-resolution T1-weighted magnetization-prepared gradient-echo images (MPRAGE) were acquired using the following parameters: repetition time (TR) = 2300 ms, echo time (TE) = 2.03 ms, flip angle (FA) = 9 deg, 1 mm^3^ isotropic voxel resolution and a Field of View (FoV) = 256×240 mm^2^ and 176 contiguous slices. Cortical and subcortical structures were segmented on each MPRAGE image using FreeSurfer v6.0.0 (http://surfer.nmr.mgh.harvard.edu/). Total brain volume, total grey matter (GM) volume, total white matter (WM) volume, total ventricular volume, and hippocampal volume were extracted from each scan.

Diffusion weighted images (DWI) were acquired using an echo planar sequence with 60 gradient directions with b-values ranging from 350 to 1600 mm/s, one unweighted B0 image, TR = 8500 ms, TE = 96 ms, 2 mm^3^ voxel resolution, 63 slices, FOV = 192 mm. DWI were corrected for B0 field inhomogeneity, Gibbs artifacts and eddy-current distortions using MRtrix 3 and FSL 5·. (http://fsl.fmrib.ox.ac.uk). Fractional anisotropy (FA) and mean diffusivity (MD) were computed by fitting the diffusion tensor to DWI data using FSL FDT. The B0 image was linearly co-registered to the MPRAGE. The inverse transformation was used to map the GM and WM masks into DWI space. FA and MD were extracted within both GM and WM global and regional masks.

All scans and processed data were quality controlled by an experienced researcher (RRG) to reduce the impact of head motion and other artifacts. Two participants were excluded due to the presence of a brain tumor. Two MPRAGE and three DWI scans were removed due to motion. Six T1-weighted and nine DWI outliers, defined as images with a metric (i.e. volume, FA, and MD) exceeding two standard deviations from the mean, were also excluded from further analyses. Groups remained balanced for age, gender and education after the exclusions.

### Biomarkers of immunosenescence

Blood specimens was collected in BD Vacutainer® CPT™ Mononuclear Cell Preparation Tubes (BD Biosciences, USA). The peripheral blood mononuclear cell (PBMC) layer was extracted and washed twice with Phosphate-buffered saline (PBS) containing 5% fetal bovine serum (FBS) (Gibco, USA). PBMCs were counted and cryopreserved in 90% FBS with 10% dimethyl sulfoxide (DMSO) (MP Biomedicals, USA) and stored at -80° C before being transferred to liquid nitrogen the following day. Cryopreserved PBMCs were thawed with the aid of cryothaw devices. Uncapped cryovials were fitted into cryothaw devices (Medax International, USA) that facilitated the transfer of cells into 15 ml conical tubes (containing warm Roswell Park Memorial Institute medium, RPMI with 10% FBS) via centrifugation at 400g for 5 minutes. Cells were subsequently washed with cold media and resuspended for counting using the MACSQuant Analyzer 8 (Miltenyi Biotec, Germany). One million cells were aliquoted into 96-well plates for staining with the antibody cocktail listed in Supplementary Table 2; the Thermo Fisher Live/Dead© fixable dye was added to the cocktail to distinguish live and dead cells. PBMCs were incubated with the mixture in the dark for 20 minutes at 4° C. Next, cells were washed twice before resuspension in Fluorescence activated cell sorting (FACS) buffer and analysed on the LSRFortessa (BD, USA). Data generated by flow cytometry were analysed by the Flowjo© software (Tree Star, Inc., USA). Events were gated by forward and side scatter followed by subset-specific marker expression to identify specific immune subsets.

### Biomarkers of oxidative damage

Plasma allantoin and urinary 8-hydroxyguanosine (8OHG) and 8-hydroxy-2' -deoxyguanosine (8OHdG) were quantified using liquid chromatography mass spectrometry (LC-MS/MS) as adapted from Cheah et al [36]. Briefly, for allantoin, 10μl of plasma were mixed with 100μl methanol containing allantoin-^15^N_4_, overnight at -20° C. Supernatants were dried under a stream of N_2_ gas and resuspended in 100μl deionised water. Samples were transferred to silanized sample vials for quantification. For 8OHG and 8OHdG, 500μl of urine samples containing 8OHdG-^13^C^15^N_2_ internal standards were clarified by solid phase extraction (Clean Screen FASt; UCT) according to manufacturer’s protocol. Eluted samples were transferred to silanized sample vials for quantification. LC-MS/MS was performed using an Agilent 1290 LC coupled to a 6460 triple quadrupole mass spectrometer (Agilent Technologies). 5μl of samples were injected onto a Hypercarb (5μm, 100x4·6mm; Thermo Scientific) for allantoin using gradient elution from 90% of 0·1% formic acid (A) to 90% acetonitrile + 0·1% formic acid (B) over 8·5 min, or Accucore PFP (2·6μm, 100x2·1mm; Thermo Scientific) for 8OHG and 8OHdG using a gradient elution from 98% A to 95% B over 10 min. MS was carried out under positive, electrospray ionization in multiple reaction monitoring mode with parameters set according to Cheah et al [36]. Quantification was carried out using a calibration curve with no less than 8 standard concentrations and adjusted as a ratio of the heavy labelled internal standards.

### Cognitive outcomes

We used a standard neuropsychological test battery to assess cognitive changes following the interventions. The tests in this battery were the Rey Auditory Verbal Learning Test (RAVLT), Digit Span, Block Design, Color Trails Test (CTT) 1 and 2, Symbol Digit Modality Test (SDMT) written and oral version, and Boston Naming Test (BNT). Ten test scores were produced: (1) RAVLT immediate recall; (2) RAVLT delayed recall; (3) Digit Span forward; (4) Digit Span backward; (5) Block Design; (6) CTT-1; (7) CTT-2; (8) SDMT written; (9) SDMT oral; (10) BNT score. The primary outcome of this trial is the change in cognitive function measured by the CCTS. The CCTS is the average of 10 Z-scores standardized to the baseline mean and standard deviation of all trial participants (Supplementary Table 1, Supplementary Data Additional Tables and Figures), with higher sores representing better cognitive function.

$$CCTS=\frac{\sum_{i=1}^{10}Zi}{10}$$

All the neuropsychological tests have been used in previous studies in Singapore [37, 38]. The CCTS was negatively correlated with age in a linear manner (Supplementary Figure 1, Supplementary Data Additional Tables and Figures), indicating that it is a good measure of age-related cognitive decline.

### Randomization and masking

The randomization list was prepared by an independent statistician from the Singapore Clinical Research Institute (SCRI) using permuted block randomization and a 1:1 allocation ratio. The block length was not made known to the clinical investigators or site personnel. Opaque, sequentially numbered and sealed envelopes were prepared by the independent statistician. Trial coordinator and trial Principal Investigator (LF) opened the randomization envelopes in sequential order to determine treatment allocation. Outcome assessors and laboratory staff were blinded to allocation status of trial participants.

### Statistical analyses

We conducted analyses using intention-to-treat principles on available data from all participants according to their allocation. We produced descriptive statistics for various characteristics of the study samples at baseline. Between-group comparisons were conducted using two-sample *t*-tests on absolute and percentage changes of CCTS from baseline. For any positive finding, we further produced adjusted estimates controlling for baseline CCTS, in view of the observed difference on CCTS at baseline (though not statistically significant with formal testing). We also compared between-group differences on CCTS at 12 and 24 months using two-sample *t* tests. A LME model was conducted to examine the main effect of time and group allocation and the interaction of time*allocation on CCTS, accounting for repeated measurements on each individual. We then fitted a series of LME models to CCTS and each brain metric and biological marker of aging. Fixed effects included time, intervention group, age, gender and years of education. The participant’s intercept and slope were considered as random effects. Interaction terms between the variables were initially included in the models, but were discarded in further analyses as no statistically significant interactions were found in any of the models. Effect sizes were calculated as: $|({\bar{x}}_{Choral}-{\bar{x}}_{HEP})|\sigma .$ All statistical analyses were conducted using IBM SPSS version 25.0 and Mat lab.

### Sample size

This interim analysis using the first cycle of intervention arms allows for the estimation of the sample size required to observe a significant change in the CCTS primary outcome variable. Assuming a two-sided type I error rate of 0.05 and statistical power of 80%, using the estimate of effect size of 0.12 (SD 0.35) of the change in CCTS over 24 months gives a sample size of 135 per intervention arms.

## Supplementary Material

Supplementary Methods

Supplementary Figures

Supplementary Tables
