# Supplementary material for: Effects of choral singing versus health education on cognitive decline and aging: a randomized controlled trial
Source: Aging (Albany NY). 2020 Dec 18;12(24):24798–816. doi: 10.18632/aging.202374 (PMC7803497; doi:10.18632/aging.202374)
Supplement: Supplementary Methods [file aging-12-202374-s001.pdf]

SUPPLEMENTARY METHODS

MRI acquisition and processing

High-resolution T1-weighted magnetization-prepared gradient-echo images (MPRAGE) were acquired using the following parameters: repetition time (TR) = 2300 ms, echo time (TE) = 2·03 ms, flip angle (FA) = 9 deg, 1 mm^3 isotropic voxel resolution and a Field of View (FoV) = 256×240 mm^2 and 176 contiguous slices. Cortical and subcortical structures were segmented on each MPRAGE image using FreeSurfer v6·0·0 (http://surfer.nmr.mgh.harvard.edu/). Total brain volume, total grey matter (GM) volume, total white matter (WM) volume, total ventricular volume, and hippocampal volume were extracted from each scan.

Diffusion weighted images (DWI) were acquired using an echo planar sequence with 60 gradient directions with b-values ranging from 350 to 1600 mm/s, one unweighted B0 image, TR = 8500 ms, TE = 96 ms, 2 mm^3 voxel resolution, 63 slices, FOV = 192 mm. DWI were corrected for B0 field inhomogeneity, Gibbs artifacts and eddy-current distortions using MRtrix 3 and FSL 5·0 (http://fsl.fmrib.ox.ac.uk). Fractional anisotropy (FA) and mean diffusivity (MD) were computed by fitting the diffusion tensor to DWI data using FSL FDT. The B0 image was linearly co-registered to the MPRAGE. The inverse transformation was used to map the GM and WM masks into DWI space. FA and MD were extracted within both GM and WM global and regional masks.

All scans and processed data were quality controlled by an experienced researcher to reduce the impact of head motion and other artifacts. Two participants were excluded due to the presence of a brain tumor. Two MPRAGE and three DWI scans were removed due to motion. Six T1-weighted and nine DWI outliers, defined as images with a metric (i.e. volume, FA, and MD) exceeding two standard deviations from the mean, were also excluded from further analyses. Groups remained balanced for age, gender and education after the exclusions.

Biomarkers of immunosenescence

Blood was collected in BD Vacutainer® CPT™ Mononuclear Cell Preparation Tubes (BD Biosciences, USA). The peripheral blood mononuclear cell (PBMC) layer was extracted and washed twice with Phosphate-buffered saline (PBS) containing 5% fetal bovine serum (FBS) (Gibco, USA). PBMCs were counted and cryopreserved in 90% FBS with 10% dimethyl sulfoxide (DMSO) (MP Biomedicals, USA) and stored at -80° C before being transferred to liquid nitrogen the following day. Cryopreserved PBMCs were thawed with the aid of cryothaw devices. Uncapped cryovials were fitted into cryothaw devices (Medax International, USA) that facilitated the transfer of cells into 15 mL conical tubes (containing warm Roswell Park Memorial Institute medium, RPMI with 10% FBS) via centrifugation at 400g for 5 minutes. Cells were subsequently washed with cold media and resuspended for counting using the MACSQuant Analyzer 8 (Miltenyi Biotec, Germany). One million cells were aliquoted into 96-well plates for staining with the antibody cocktail listed in Supplementary Table 2; the Thermo Fisher Live/Dead© fixable dye was added to the cocktail to distinguish live and dead cells. PBMCs were incubated with the mixture in the dark for 20 minutes at 4° C. Next, cells were washed twice before resuspension in Fluorescence activated cell sorting (FACS) buffer and analysed on the LSRFortessa (BD, USA). Data generated by flow cytometry were analysed by the Flowjo© software (Tree Star, Inc., USA). Events were gated by forward and side scatter followed by subset-specific marker expression to identify specific immune subsets.

Biomarkers of oxidative damage

Plasma allantoin and urinary 8-hydroxyguanosine (8OHG) and 8-hydroxy-2' -deoxyguanosine (8OHdG) were quantified using liquid chromatography mass spectrometry (LC-MS/MS) as adapted from Cheah et al. Briefly, for allantoin, 10µl of plasma were mixed with 100µl methanol containing allantoin-$^{15}N_4$, overnight at -20° C. Supernatants were dried under a stream of N_2 gas and resuspended in 100µl deionised water. Samples were transferred to silanized sample vials for quantification. For 8OHG and 8OHdG, 500µl of urine samples containing 8OHdG-$^{13}C^{15}N_2$ internal standards were clarified by solid phase extraction (Clean Screen FASt; UCT) according to manufacturer's protocol. Eluted samples were transferred to silanized sample vials for quantification. LC-MS/MS was performed using an Agilent 1290 LC coupled to a 6460 triple quadrupole mass spectrometer (Agilent Technologies). 5µl of samples were injected onto a Hypercarb (5µm, 100x4·6mm; Thermo Scientific) for allantoin using gradient elution from 90% of 0·1% formic acid (A) to 90% acetonitrile + 0·1% formic acid (B) over 8·5 min, or Accucore PFP (2·6µm, 100x2·1mm; Thermo Scientific) for 8OHG and 8OHdG using a gradient elution from 98% A to 95% B over 10 min. MS was carried out under positive, electrospray ionization in multiple reaction monitoring mode with parameters set

according to Cheah et al. Quantification was carried out using a calibration curve with no less than 8 standard concentrations and adjusted as a ratio of the heavy labelled internal standards.
