# Supplementary material for: Effects of choral singing versus health education on cognitive decline and aging: a randomized controlled trial
Source: Aging (Albany NY). 2020 Dec 18;12(24):24798–816. doi: 10.18632/aging.202374 (PMC7803497; doi:10.18632/aging.202374)
Supplement: Supplementary Figures [file aging-12-202374-s002.pdf]

SUPPLEMENTARY FIGURES

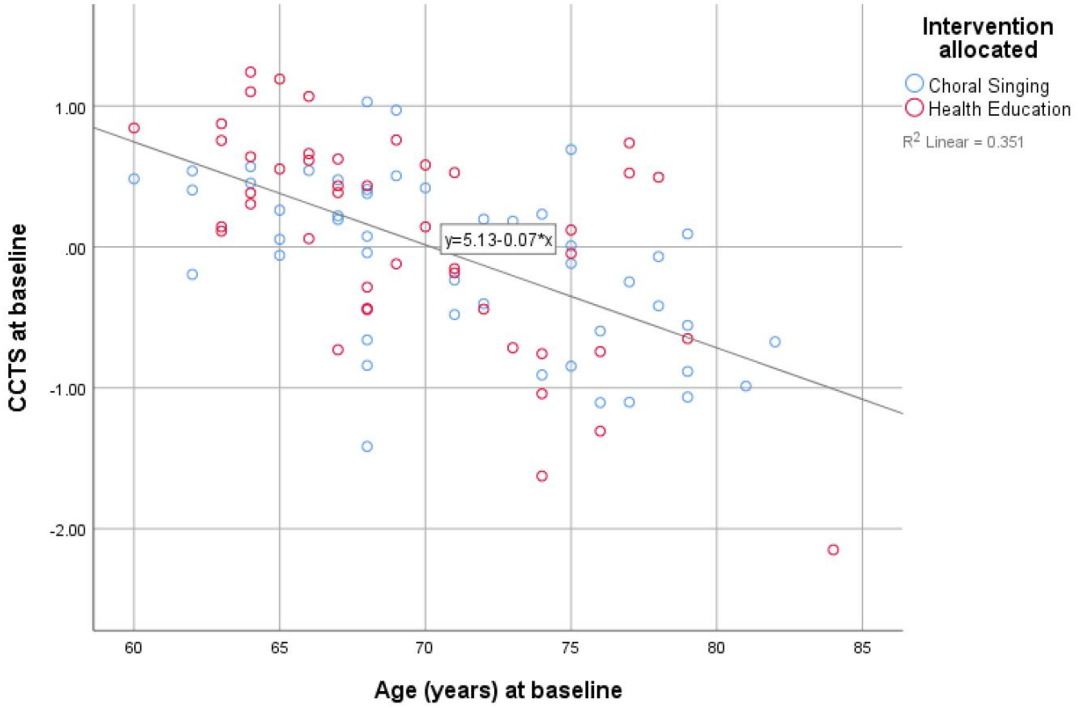

Supplementary Figure 1. Baseline CCTS scores: correlation with age (years).

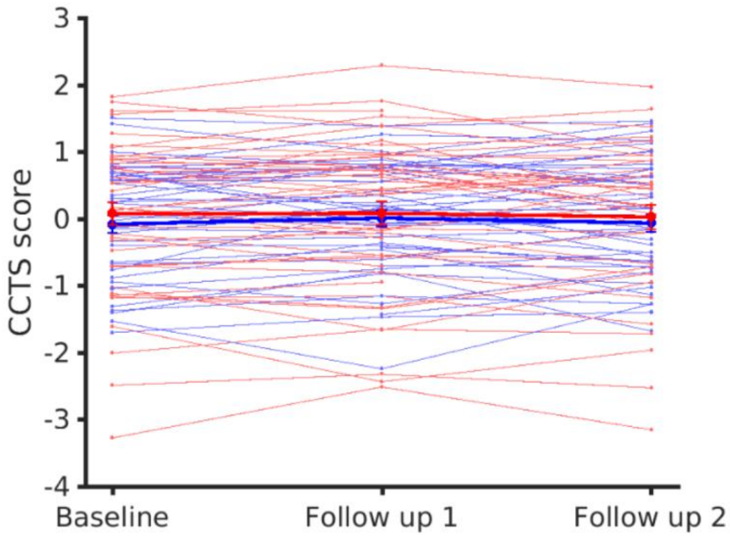

Supplementary Figure 2. CCTS of 47 CSI and 44 HEP participants across time. Thin lines represent individual participants whereas thick lines illustrate the average of the choral singing (blue) and health education (red) groups. Values are normalized to sample average baseline. Error bars represent the standard error of the mean.

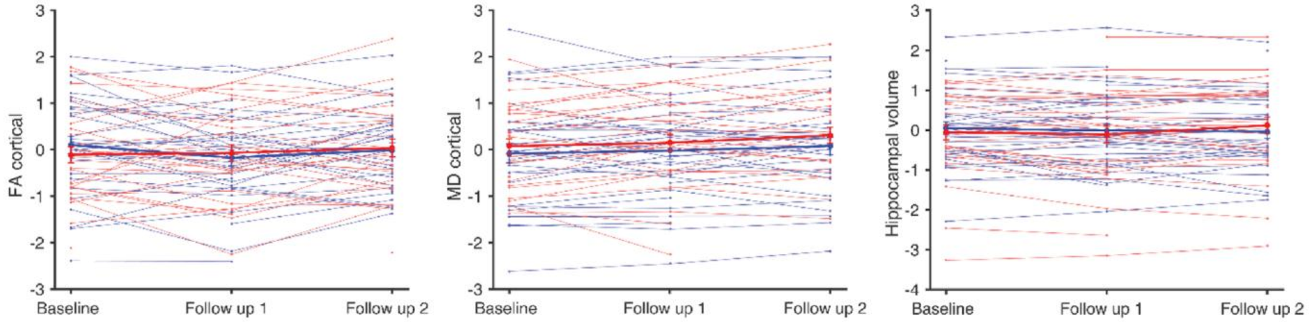

Supplementary Figure 3. Additional brain MRI metrics of each participant across time. Thin lines represent individual participants whereas thick lines illustrate the average of the Choral (blue) and HEP (red) groups. Values are normalized to each individual baseline. Error bars represent the standard error of the mean.

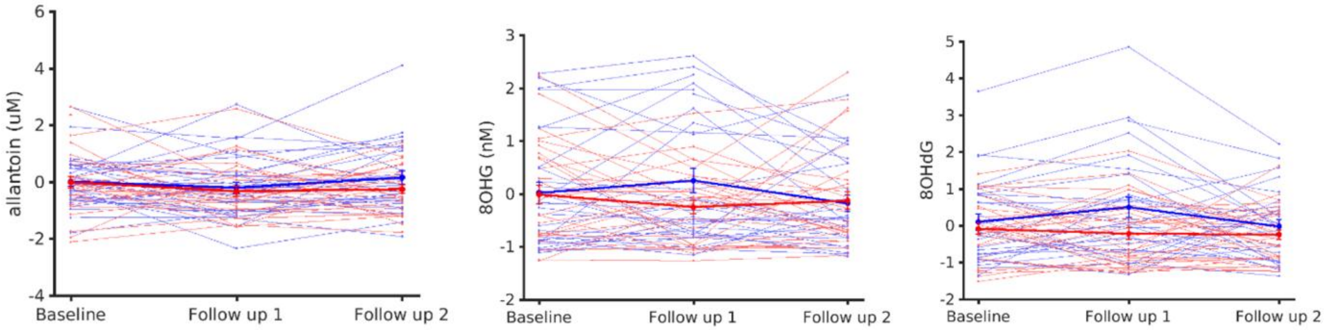

Supplementary Figure 4. Markers of oxidative stress for each participant across time. Thin lines represent individual participants whereas thick lines illustrate the average values of the choral singing (blue) and health education (red) groups. Values are normalized to sample average baseline. Error bars represent the standard error of the mean.

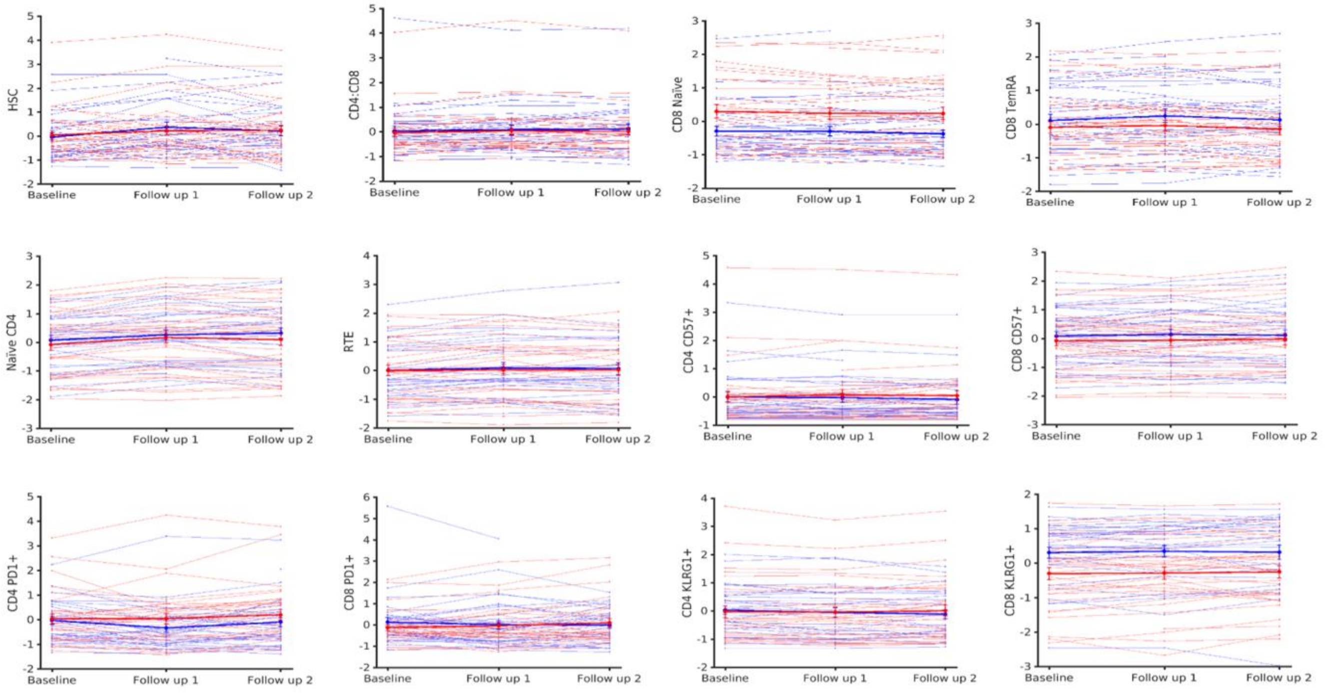

Supplementary Figure 5. Frequency of immune subsets for monitoring immunosenescence of 26 CSI and 32 HEP participants across time. Hematopoietic stem cells (HSC), CD4:CD8 ratios, naïve and TEMRA CD8 T-cells, Naïve CD4 T-cells, CD4 Recent Thymic Emigrants (RTEs), and T-cells expressing exhaustion and senescence markers (CD57, PD1, KLRG1) are shown. Thin lines represent individual participants whereas thick lines illustrate the average values of the Choral (blue) and HEP (red) groups. Values are normalized to the population mean. Error bars represent the standard error of the mean.
