# Supplementary material for: Effects of choral singing versus health education on cognitive decline and aging: a randomized controlled trial
Source: Aging (Albany NY). 2020 Dec 18;12(24):24798–816. doi: 10.18632/aging.202374 (PMC7803497; doi:10.18632/aging.202374)
Supplement: Supplementary Tables [file aging-12-202374-s003.pdf]

SUPPLEMENTARY TABLES

Supplementary Table 1. Descriptive statistics of the 10 cognitive test scores at baseline.

Test score	N	Minimum	Maximum	Mean	SD
RAVLT immediate recall	93	23	71	46.63	10.632
RAVLT delayed recall	93	0	15	9.98	3.043
Digit Span forward	93	5	16	10.65	2.636
Digit Span backward	93	2	14	6.06	2.151
Block Design	93	3	54	27.58	9.846
CTT 1	93	33	214	69.01	29.991
CTT 2	93	63	315	134.05	47.183
SDMT written	93	6	59	31.97	11.871
SDMT oral	93	4	67	39.13	13.431
BNT	93	10	30	22.10	5.351

Supplementary Table 2. List of antibodies used in T-cell phenotyping panel.

Antibody	Fluorochrome	Clone	Company	Cat
CD3	BV570	UCHT1	Biolegend	300436
CD4	A700	OKT4	Biolegend	317426
CD8	BV650	RPA-T8	BD	563821
CD127	APC	A019D5	Biolegend	351316
CD25	PE/Cy7	M-A251	Biolegend	356108
CD27	APC/Cy7	M-T271	Biolegend	356424
CD45RO	BV785	UCHL1	Biolegend	304234
CD31	BV605	WM59	Biolegend	303122
CD57	PerCP/Cy5.5	HNK-1	Biolegend	359622
KLRG1	PE-Vio615	REA261	Miltenyi Biotec	130-108-366
PD1	BV421	MIH4	BD	564323
CD95	PE/Cy5	DX2	eBioscience	15-0959-42
CD34	PE	563	BD	550761
Vd1	FITC	REA173	Miltenyi Biotec	130-100-532
L/D	Aqua		Life Tech	L34966
